# Supplementary material for: Warming drives dissolved organic carbon export from pristine alpine soils
Source: Nat Commun. 2024 Apr 25;15:3522. doi: 10.1038/s41467-024-47706-6 (PMC11045798; doi:10.1038/s41467-024-47706-6)
Supplement: Supplementary file 1 — Supplementary information [file 41467_2024_47706_MOESM1_ESM.pdf]

Supplementary Information

Warming drives dissolved organic carbon export from pristine alpine soils

Andrew R. Pearson[1, 2*]; Bethany R.S. Fox[3]; John C. Hellstrom[4]; Marcus J. Vandergoes[5]; Sebastian F.M. Breitenbach[6]; Russell N. Drysdale[4]; Sebastian N. Höpker[1]; Christopher T. Wood[1,5]; Martin Schiller[7] Adam Hartland[1,8*].

[1]Environmental Research Institute, School of Science, Faculty of Science and Engineering, University of Waikato, Kirikiriroa Hamilton, Waikato, Aotearoa New Zealand.

[2]Institute of Environmental Science and Research (ESR), Ōtautahi Christchurch, Aotearoa New Zealand.

[3]Department of Biological and Geographical Sciences, University of Huddersfield, United Kingdom.

[4]School of Geography, Earth and Atmospheric Sciences, University of Melbourne, Victoria, Australia.

[5]GNS Science, *Te Awa Kairangi ki Tai* Lower Hutt, Aotearoa New Zealand.

[6]Department of Geography and Environmental Sciences, Northumbria University, United Kingdom.

[7]Centre for Star and Planet Formation, Globe Institute, University of Copenhagen, Copenhagen, Denmark.

[8]Lincoln Agritech Ltd, Ruakura, Kirikiriroa Hamilton, Waikato, Aotearoa New Zealand.

*Corresponding authors: Andrew.Pearson@esr.cri.nz; Adam.Hartland@waikato.ac.nz

Cave sites

Supplementary Table 1- Local climate, vegetation, and soil characteristics. Soils described using the New Zealand Soil Classification[1].

Cave site	Location	Local Climate	Local vegetation	Local soil description
**Hodge Creek Cave**	**Lat:** 41.19 °S. **Long:** 172.72 °E. **Altitude**: 940 m. Kahurangi NP, Nelson, NW Te Waipounamu.	Surface MAT: ~10 °C. Annual Precipitation: 1,434 mm	Mature beech forest, mosses.	Heavily weathered Oligocene limestone, incised by deep grykes filled with litter organic (LO) soils, which transition to orthic gley (GO) due to waterlogging (Fe reduction) (Supplementary Figure 1)
**Dave's Cave**	**Lat:** 45.39 °S. **Long:** 167.59 °E. **Altitude:** 1,450 m. Fiordland NP, Southland, SW Te Waipounamu.	Surface MAT: ~6 °C Annual Precipitation: 3,786 mm (Lake Manapouri).	Located above the treeline (beech). Thick tussock, grasses, native alpine plants.	Water-logged, organic rich. Soils had light brown A horizons at 20 cm. Characteristic Fe staining indicating Fe reduction and oxidation (Supplementary Figure 2).

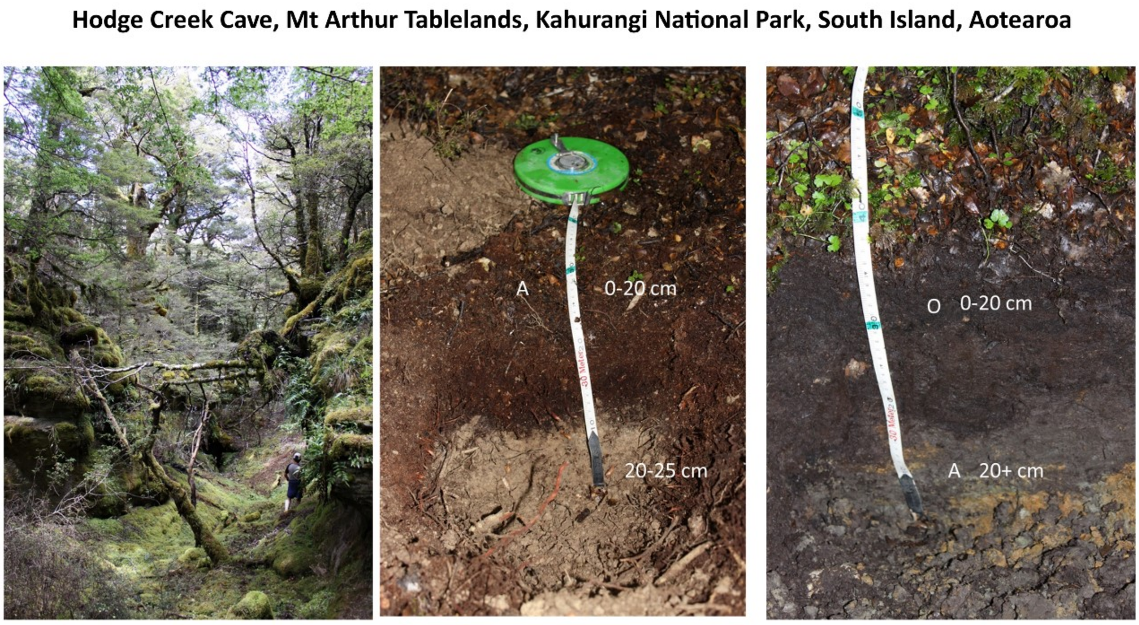

Supplementary Figure 1- Environmental setting above Hodge Creek Cave. Vegetation and soil horizons above Hodge Creek Cave.

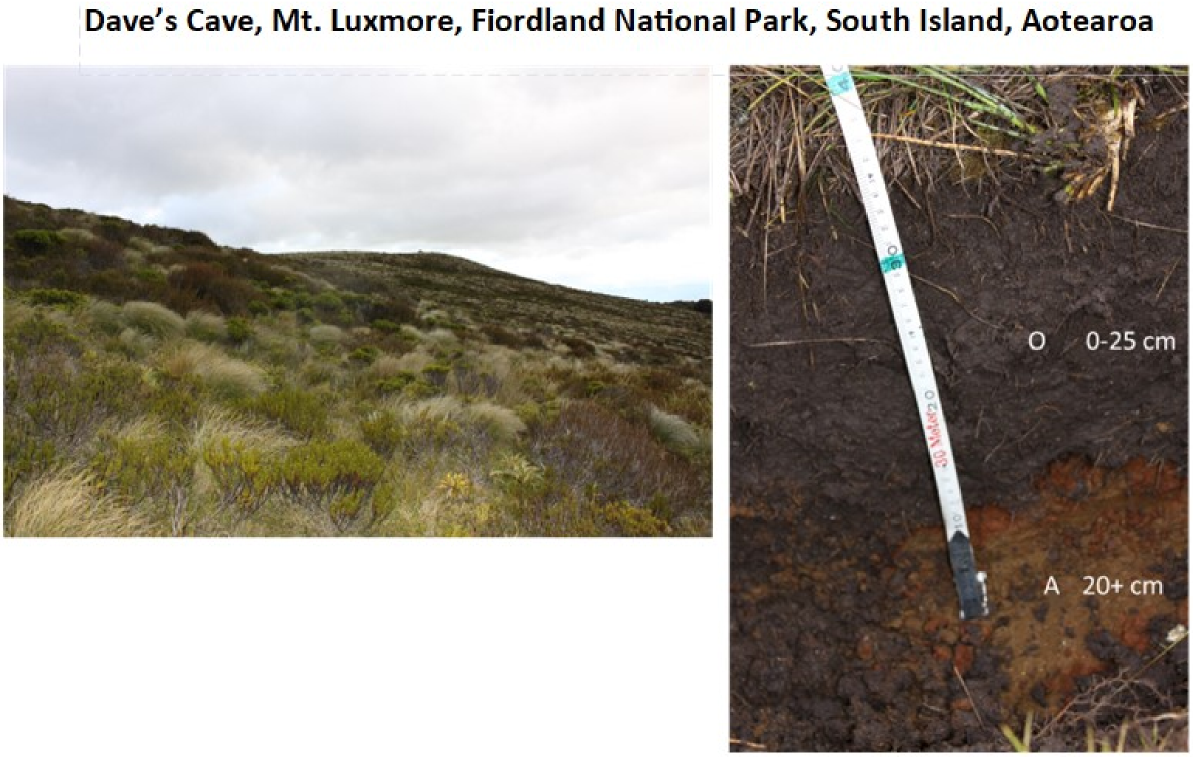

Supplementary Figure 2- Environmental setting above Dave's Cave. Vegetation above Dave's Cave and soil horizons above Dave's Cave.

U–Th geochronology of flowstones

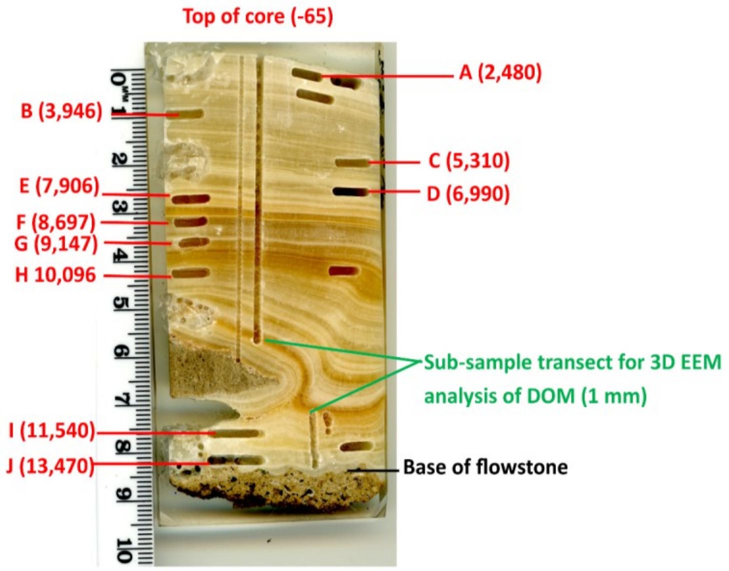

Supplementary Figure 3- Face of Hodge Creek flowstone core (HC15-2). Red labels are U–Th sub-samples with age in parentheses (Supplementary Table 2).

Supplementary Table 2- U–Th ages for Hodge Creek flowstone core (HC15-2)

U–Th Sample ID	Depth along growth-axis (mm)	Age (years)	2 σ (years)
Top of core-2015	0	-65	0
HC15-2 A	2	2480	220
HC15-2 B	12.4	3946	130
HC15-2 C	20.9	5310	110
HC15-2 D	27.85	6990	142
HC152 E	30.75	7906	260
HC152 F	35.05	8697	174
HC152 G	38.7	9147	115
HC152 H	45.85	10096	55
HC15-2 I	55.7	11540	113
HC15-2 J	64.95	13470	140

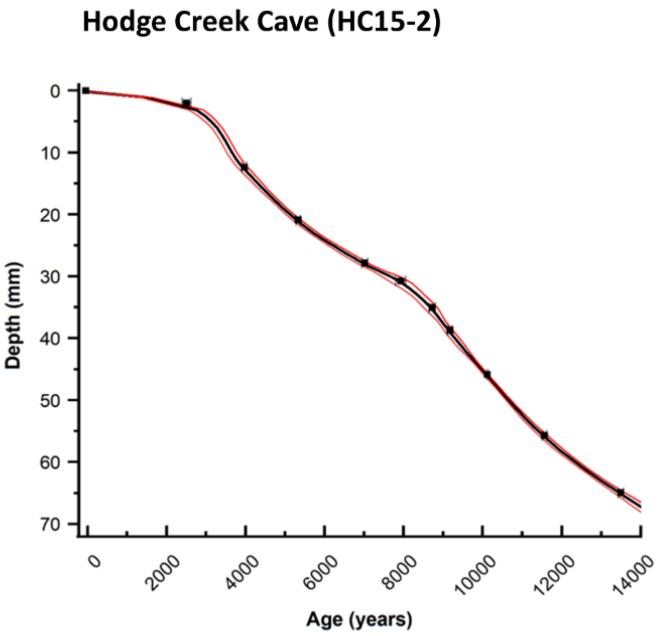

Supplementary Figure 4- Age-depth model for Hodge Creek core HC15-2. Red lines show 95 % confidence intervals.

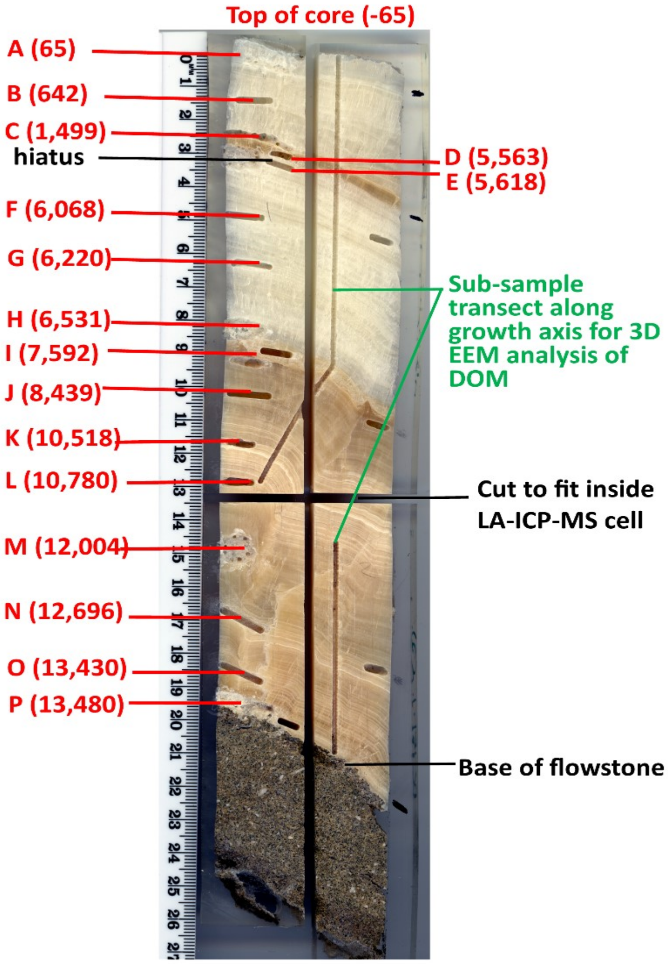

Supplementary Figure 5- Face of Dave's Cave flowstone core (DC15-1). Red labels show U/Th sub-samples with age in parentheses. (Supplementary Table 3). Note: to fit into the LA-ICPMS cell, flowstone sample was cut into four sections.

Supplementary Table 3- U–Th ages for Dave's Cave flowstone core (DC15-1).

U–Th Sample ID	Depth along growth-axis (mm)	Age (years)	2 σ (years)
Top of core- 2015	0	-65	0
DC15-1 A	3	65	94
DC15-1 B	16.5	642	33
DC15-1 C	24.8	1499	126
DC15-1 D	28.45	5563	106
DC15-1 E	33.85	5618	108
DC15-1 F	49.85	6068	50
DC15-1 G	62.75	6220	52
DC15-1 H	85.6	6531	55
DC15-1 I	90.9	7592	74
DC15-1 J	105.7	8439	98
DC15-1 K	125.7	10518	191
DC15-1 L	134.2	10780	200
DC15-1 M	142.8	12004	152
DC15-1 N	172.45	12696	178
DC15-1 O	189.7	13430	230
DC15-1 P	196.1	13480	1450

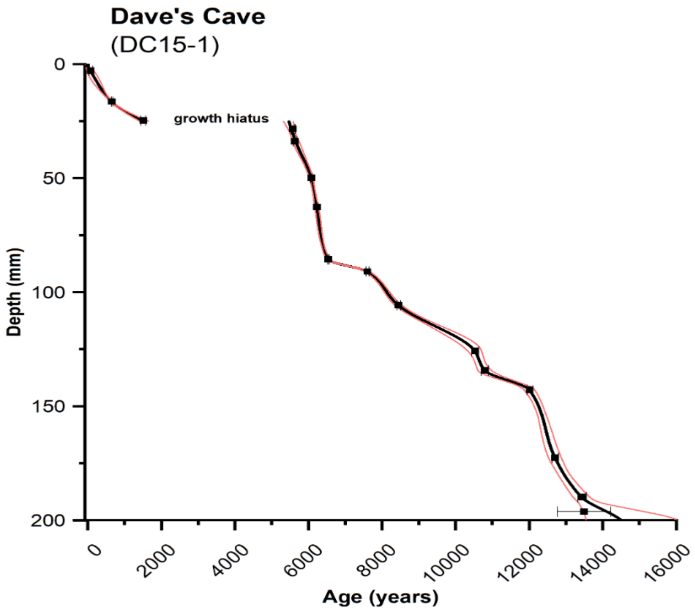

Supplementary Figure 6- Age Depth model for Dave's Cave core 'DC15-1'. Red lines show 95% confidence intervals.

Adelaide Tarn age-dating

At Adelaide Tarn, two overlapping sediment cores were collected using a piston corer and dated as part of a previous study[2]. The Bayesian age-model of the core was based on 16 radiocarbon dates utilising the SHCal13 calibration curve[2,3].

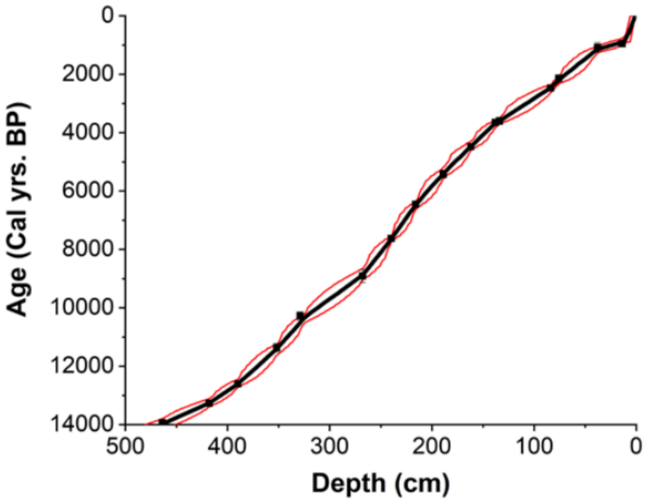

Supplementary Figure 7- Bayesian age model based on AMS radiocarbon dates from Adelaide Tarn reproduced from Jara et al., (2015). The model was developed using BACON[4] in R software. Calibration was based on the SHCal13 calibration curve (Hogg et al., 2013) with a calibration created using CALIB 6.01[5]. The image shows a modelled weighted mean age (black line) and the 95% confidence intervals (red lines).

Total organic carbon (TOC) and Fourier-Transform Infrared spectroscopy (FTIRS) analysis of lake sediments

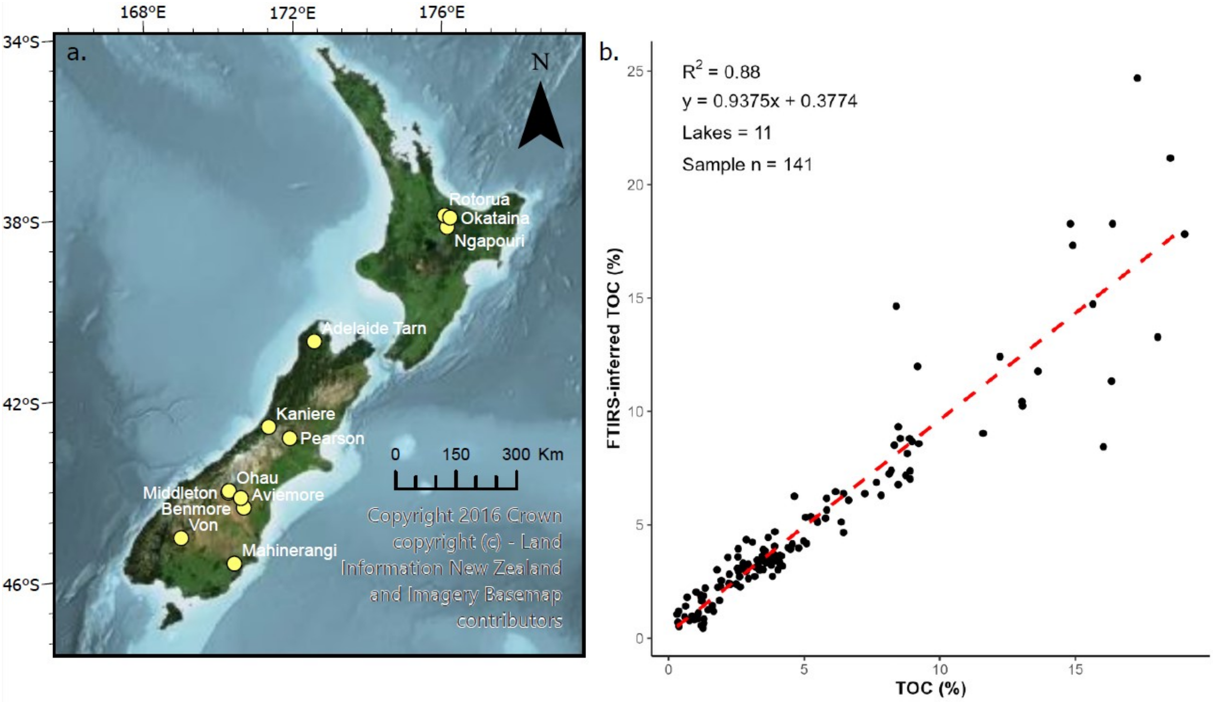

Supplementary Figure 8- TOC and FTIRS analysis of lake sediments from eleven lakes used for reconstructing Adelaide Tarn TOC (a) Location of lakes within Aotearoa New Zealand. This basemap includes Toitū Te Whenua Land Information New Zealand data which are licensed by Toitū Te Whenua Land Information New Zealand for re-use under the Creative Commons Attribution 4.0 International licence. **(b.)** Relationship between conventionally measured TOC and FTIRS-inferred TOC concentrations in sediments from eleven

Aotearoa lakes[6]. A partial-least squares regression model from these data was used to infer sediment TOC concentrations through time in Adelaide Tarn.

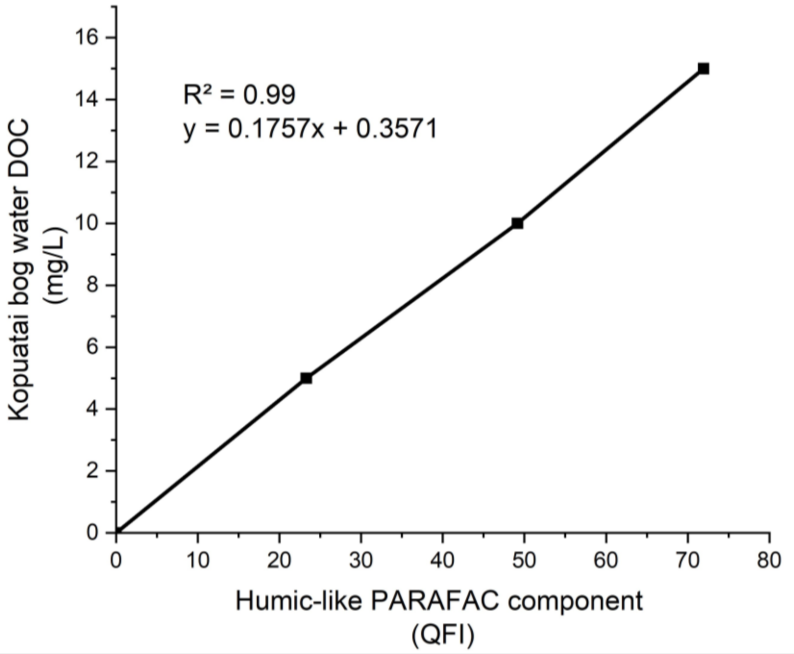

Supplementary Figure 9- Fluorescence intensity of humic-like PARAFAC component against DOC concentration in water from Kopuatai bog.

Calibration of flowstone humic-like DOC concentrations using natural DOC standards

Supplementary Table 4- Modern dripwater humic-like DOC concentrations at Dave's Cave and Hodge Creek Cave.

**Site**	**Contemporary DOC concentrations (mg C L^{-1})** Mean | Min | Max | St. dev. | n
**Hodge Creek Cave**	4.17 | 2.87 | 7.21 |1.96| 5
**Dave's Cave**	1.69 | 1.12 | 2.27 | 0.93 | 2

Supplementary References

1 Hewitt, A. E. New Zealand soil classification. *Landcare research science series* (2010).

2 Jara, I. A. *et al.* Pollen–climate reconstruction from northern South Island, New Zealand (41°S), reveals varying high- and low-latitude teleconnections over the last 16 000 years. *Journal of Quaternary Science* **30**, 817-829, doi:10.1002/jqs.2818 (2015).

3 Hogg, A. G. *et al.* SHCal13 Southern Hemisphere calibration, 0–50,000 years cal BP. *Radiocarbon* **55**, 1889-1903 (2013).

4 Blaauw, M. & Christen, J. A. Flexible paleoclimate age-depth models using an autoregressive gamma process. *Bayesian analysis* **6**, 457-474 (2011).

5 Stuiver, M. & Reimer, P. J. Extended 14 C data base and revised CALIB 3.0 14 C age calibration program. *Radiocarbon* **35**, 215-230 (1993).

6 Pearson, A. R., Fox, B. R., Vandergoes, M. J. & Hartland, A. The sediment fluorescence–trophic level relationship: using water-extractable organic matter to assess past lake water quality in New Zealand. *New Zealand Journal of Marine and Freshwater Research* **56**, 213-233 (2022).
